# Safety and efficacy of radiofrequency ablation for papillary thyroid carcinoma adjacent to the thyroid capsule

**DOI:** 10.3389/fonc.2026.1820010

**Published:** 2026-08-05

**Authors:** Yuxi Liu, Yunfeng Wu, Qing Xia, Miaomiao Wang, Ying Che

**Affiliations:** 1 Department of Ultrasound, Affiliated Hospital of Shandong Second Medical University, Shan Dong, Weifang, China; 2 Department of Ultrasound, The First Affiliated Hospital of Dalian Medical University, Liaoning, Dalian, China; 3 School of Teacher Education, Weifang University, Weifang, China

**Keywords:** efficacy, radiofrequency ablation, safety, thyroid neoplasms, ultrasound guidance

## Abstract

**Background:**

Ultrasound-guided radiofrequency ablation (RFA) has become an established minimally invasive treatment for selected patients with low-risk papillary thyroid carcinoma (PTC). However, the safety and efficacy of RFA for PTCs located immediately adjacent to the thyroid capsule (≤2 mm) remain insufficiently characterized because of the increased risk of thermal injury to adjacent critical structures. This study aimed to evaluate the safety and oncologic outcomes of ultrasound-guided RFA for solitary T1N0M0 PTC in this anatomically challenging setting.

**Methods:**

This single-center retrospective study included 303 consecutive patients with solitary PTC located within 2 mm of the thyroid capsule who underwent ultrasound-guided RFA between June 2015 and December 2023. All diagnoses were confirmed by fine-needle aspiration, and all patients declined surgical treatment. Tumors were classified as T1a (n = 263) or T1b (n = 40). The primary efficacy endpoints were technical success and complete tumor disappearance. The primary safety endpoint was the incidence of procedure-related complications. Disease progression was assessed as a secondary endpoint. Patients underwent serial ultrasonographic follow-up according to a predefined protocol.

**Results:**

Technical success was achieved in all patients (100%, 303/303). After a median follow-up of 36 months, complete tumor disappearance of the treated tumor was observed in 294 patients (97.0%). The complete disappearance rate was higher in the T1a group than in the T1b group (98.1% [258/263] vs 90.0% [36/40]), and the difference was statistically significant (P <0.001). Disease progression occurred in five patients (1.65%), all in the T1a group, including four cases of newly developed primary thyroid carcinoma and one case of cervical lymph node metastasis. Procedure-related complications occurred in 10 patients (3.3%), all of whom experienced transient hoarseness that resolved spontaneously within 6 months. The complication rate was significantly higher in the T1b group than in the T1a group (10.0% [4/40] vs 2.3% [6/263]; P = .034). BRAF^V600E^ mutation status was not associated with disease progression (P = .99).

**Conclusion:**

Ultrasound-guided RFA combined with hydrodissection and the lever maneuver is an effective and generally safe minimally invasive treatment option for solitary T1N0M0 PTC located adjacent to the thyroid capsule. Careful patient selection remains essential, as T1b tumors were associated with a numerically lower complete disappearance rate and a significantly higher risk of transient hoarseness compared with T1a tumors.

## Introduction

The global incidence of thyroid cancer has increased substantially in recent decades, largely owing to the widespread application of diagnostic imaging and fine-needle aspiration biopsy (1). Papillary thyroid carcinoma (PTC) represents the most common histological subtype of thyroid cancer, followed by follicular, medullary, and anaplastic thyroid carcinomas (2). Although most PTCs exhibit indolent biological behavior and excellent disease-specific survival, surgery remains the conventional standard treatment. However, thyroidectomy is associated with potential complications, including recurrent laryngeal nerve injury, hypoparathyroidism, cervical scarring, and the requirement for lifelong thyroid hormone replacement. For patients with low-risk papillary thyroid microcarcinoma (≤1 cm) or small T1-stage PTC, immediate surgery may represent overtreatment, leading to increasing interest in active surveillance and minimally invasive treatment approaches (3).

Ultrasound-guided percutaneous RFA has been increasingly adopted as a minimally invasive treatment for benign thyroid nodules and selected low-risk PTCs. Compared with surgery, RFA offers several potential advantages, including avoidance of surgical scars, shorter procedure duration, preservation of thyroid function, and a favorable safety profile. Accumulating evidence from prospective and retrospective studies has demonstrated that RFA can achieve oncologic outcomes comparable to surgery in selected patients with T1aN0M0 and T1bN0M0 PTC, with low rates of local tumor progression, lymph node metastasis, and distant metastasis during follow-up (4–7).

Nevertheless, the application of RFA for PTCs located immediately adjacent to the thyroid capsule remains technically challenging and clinically controversial. Tumors within 2 mm of the thyroid capsule, particularly those located in the posterior or posteromedial region adjacent to the tracheoesophageal groove (the so-called “danger triangle”), are in close proximity to critical structures, including the RLN, trachea, esophagus, and parathyroid glands. Limited thermal safety margins in these locations increase the risk of thermal injury, especially RLN thermal injury (RLNTI), which may result in transient or permanent vocal cord dysfunction. Recent predictive models have identified a tumor distance to the tracheoesophageal groove of ≤2.95 mm, dorsal tumor location, and higher ablation energy as independent risk factors for RLNTI (8). In addition, subcapsular tumors may present unique technical challenges due to limited space for electrode manipulation and potential adhesion to adjacent structures, which may compromise complete ablation and increase the risk of residual or recurrent disease (5).

Several technical modifications have been proposed to improve the safety of RFA in anatomically high-risk locations. These include extensive peritracheal hydrodissection and the use of short active-tip electrodes to increase thermal control and protect adjacent structures (9, 10). However, evidence specifically evaluating RFA for PTCs located immediately adjacent to the thyroid capsule (≤2 mm) remains limited. Most previous studies have included these lesions within broader T1-stage cohorts, making it difficult to determine their specific procedural risks and oncologic outcomes.

Therefore, this study aimed to evaluate the safety and oncologic efficacy of ultrasound-guided RFA in a well-defined cohort of patients with solitary T1N0M0 PTC located within 2 mm of the thyroid capsule. We assessed technical success, complete tumor disappearance, disease progression, and procedure-related complications to clarify the feasibility of RFA in this anatomically challenging subgroup and provide evidence for individualized treatment selection.

## Methods

### Patients

**Figure 1:**
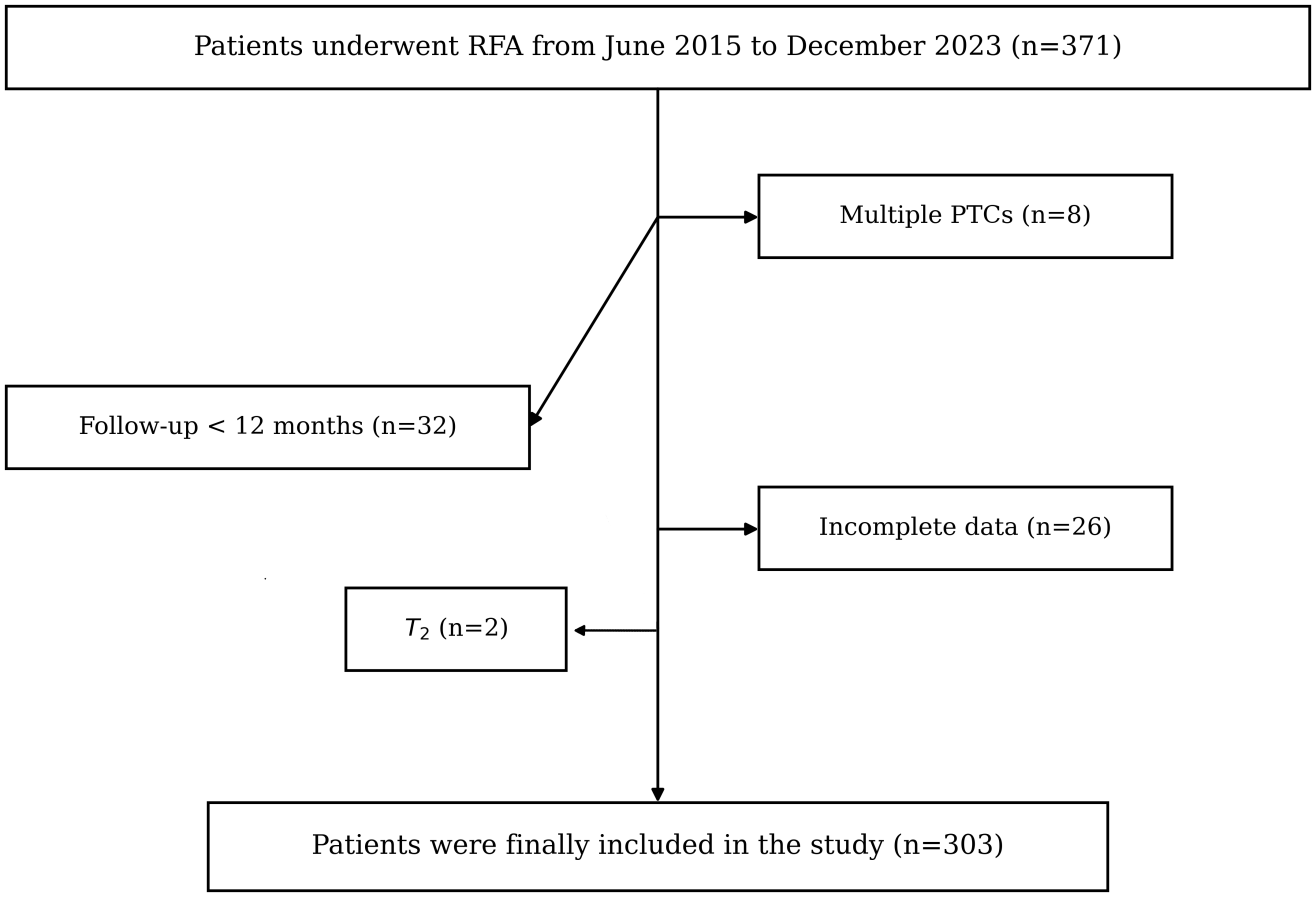
Research flowchart. RFA, radiofrequency ablation; PTC, papillary thyroid carcinoma.

This retrospective study was conducted at a single tertiary medical center and included 303 consecutive patients with solitary PTC who underwent ultrasound-guided RFA between June 2015 and December 2023 (**Figure 1**). The study population comprised 223 women and 80 men, with a median age of 43 years (range, 24–77 years). Tumor stage was classified according to the maximum tumor diameter (MD) measured by preprocedural ultrasonography, including T1a tumors (≤1 cm; n = 263) and T1b tumors (>1 cm and ≤2 cm; n = 40). The median follow-up duration was 36 months (interquartile range [IQR], 24–36 months).

The inclusion criteria were as follows: (1) cytologically confirmed PTC by fine-needle aspiration (FNA); (2) solitary thyroid tumor located within 2 mm of the thyroid capsule on preprocedural ultrasonography, without evidence of extrathyroidal extension, cervical lymph node metastasis (LNM), or distant metastasis; (3) refusal of surgical treatment or unsuitability for surgery based on individual clinical considerations; and (4) availability of complete follow-up data for at least 12 months.

The exclusion criteria were: (1) multifocal PTC; (2) imaging evidence of gross capsular invasion or invasion of adjacent anatomical structures; (3) incomplete preprocedural or postprocedural imaging data; or (4) loss to follow-up.

This study was approved by the Ethics Committee of the First Affiliated Hospital of Dalian Medical University (No. PJ-KS-KY-2022-346), which waived the requirement for informed consent. Informed consent was waived due to the retrospective nature of the study and anonymized data analysis.

### Ablation equipment and procedure

All RFA procedures were performed using a commercially available RFA system (Mianyang Lead Electronic Co., Ltd., Mianyang, China) equipped with internally cooled, straight electrodes with active-tip lengths of 7 or 10 mm. Real-time ultrasound guidance was provided using a GE Logiq E9 ultrasound system (GE Healthcare, Milwaukee, WI, USA) with a 9L linear-array transducer (frequency, 9 MHz).

Preprocedural evaluation included high-resolution neck ultrasonography, cervical computed tomography (CT), and laboratory examinations. Tumor volume was calculated using the ellipsoid formula: *V* = *πabc*/6, where *a* represents the maximum tumor diameter and *b* and *c* represent the two perpendicular diameters (11).

Patients were placed in the supine position with neck extension. Local anesthesia was achieved using 1% lidocaine. Under ultrasound guidance, a 23-gauge needle was inserted between the thyroid capsule and surrounding tissues, and 5% glucose solution was injected to create a protective fluid barrier of at least 5 mm between the tumor and adjacent critical structures (hydrodissection technique).

For tumors located within the posterior or posteromedial “danger triangle” adjacent to the trachea or esophagus, an additional lever maneuver was performed. Specifically, the RFA electrode was used to mechanically displace the tumor away from adjacent high-risk structures before energy delivery, thereby increasing the safety margin.

Ablation was initiated at a power setting of 60 W. The moving-shot technique was used to sequentially ablate the tumor. A location-specific ablation strategy was used. For tumor margins adjacent to the thyroid capsule, ablation was terminated once the transient hyperechoic zone reached the capsule to minimize thermal injury to adjacent critical structures. Conversely, where the tumor margin was surrounded by normal thyroid parenchyma, ablation was continued until the transient hyperechoic zone completely encompassed the tumor and, when anatomically feasible, extended approximately 5 mm beyond the original tumor margin to ensure an adequate ablative margin. Immediate post-ablation contrast-enhanced ultrasonography (CEUS) was performed to assess technical success. Additional ablation was performed when residual intratumoral enhancement was identified.

### Follow-up and outcome assessment

After RFA, patients underwent standardized follow-up including clinical evaluation and neck ultrasonography at 1, 3, 6, 9, and 12 months during the first year; every 6 months thereafter. Thyroid function tests and complete blood counts were obtained during follow-up visits. Cervical CT was performed annually for surveillance of distant metastasis. CEUS and repeat FNA were performed only when imaging findings suggested possible residual tumor, local recurrence, or cervical lymph node metastasis.

The primary safety endpoint was the incidence of procedure-related complications, classified according to the Society of Interventional Radiology (SIR) guidelines.

The primary efficacy endpoints were defined as follows:

Technical success: Complete absence of intratumoral enhancement on immediate post-ablation CEUS.

Complete tumor disappearance: Complete tumor disappearance of the treated lesion during follow-up, leaving only a linear hyperechoic scar at the ablation site.

Disease progression: Disease progression included local tumor progression (regrowth of the treated lesion), development of a new primary thyroid carcinoma, occurrence of cervical lymph node metastasis, or distant metastasis.

### Statistical analysis

Continuous variables are presented as medians with interquartile ranges (IQRs), and categorical variables are presented as frequencies with percentages. Changes in tumor volume over time were evaluated using the Friedman test comparing baseline volume with measurements obtained at 1, 3, 6, 9, and 12 months after RFA.

Comparisons between the T1a and T1b groups were performed using the χ² test or Fisher’s exact test for categorical variables. Continuous variables with non-normal distributions were compared using the Mann-Whitney U test. Time to complete tumor disappearance was analyzed using the Kaplan–Meier method, and differences between groups were assessed using the log-rank test.

All statistical analyses were performed using SPSS software (version 25.0; IBM Corp., Armonk, NY, USA). A two-sided P value <.05 was considered statistically significant.

## Results

### Patient and tumor characteristics

A total of 303 patients with solitary PTC were included in this study, comprising 263 patients with T1a tumors and 40 patients with T1b tumors. Baseline clinical and tumor characteristics are summarized in **Table 1**.

**Table 1:** Basic characteristics of study population (n = 303).

Variable	Total (n = 303)	T1a group (n=263)	T1b group (n=40)	P value
Age (years)	43.0 (35.0, 52.0)	43.0 (35.0, 52.0)	40.0 (36.0, 52.0)	0.726
Sex (%)				
F	223 (73.6%)	188 (71.8%)	35 (87.5%)	0.051
M	80 (26.4%)	75 (28.5%)	5 (12.5%)	
Follow-Up (months)	36.0 (24, 36)	36.0 (24.0, 36.0)	30 (24.0, 36.0)	
BRAF^V600E^ mutation (%)				0.669
Positive	68 (22.4%)	62 (23.6%)	6 (15.0%)	
Negative	235 (77.6%)	201 (76.4%)	34 (75.0%)	
Location (%)				0.065
anterior capsule	114 (37.6%)	95 (36.1%)	19 (47.5%)	
posterior capsule	111 (36.6%)	93 (35.4%)	18 (45.0%)	
isthmus	48 (15.8%)	46 (17.5%)	2 (5.0%)	
paratracheal	30 (9.9%)	29 (11.0%)	1 (2.5%)	
HT (%)				0.675
0	216 (71.3%)	187 (71.1%)	29 (72.5%)	
1	87 (28.7%)	73 (27.8%)	14 (35.0%)	
Time (s)	35.0 (25.0, 54.0)	33.0 (23.0, 46.0)	67.5 (49.3, 90.0)	<0.001

The median age was comparable between the T1a and T1b groups (43.0 years [IQR, 35.0-52.0 years] vs 40.0 years [IQR, 36.0-52.0 years], respectively). The median follow-up duration was also similar between groups (36.0 months [IQR, 24.0-36.0 months] vs 30.0 months [IQR, 24.0-36.0 months], respectively). The upper quartile coincided with the median because follow-up durations were highly clustered at 24 and 36 months.

The proportion of female patients showed a borderline difference between the T1a and T1b groups (71.5% vs 87.5%, respectively; χ² test, P = .051). Tumor location did not differ significantly between groups (P = .065). No significant differences were observed in BRAFV600E mutation status (23.2% vs 17.5%, respectively; P = .493) or the presence of Hashimoto thyroiditis (27.8% vs 35.0%, respectively; P = .675).

The median ablation time was significantly longer in the T1b group than in the T1a group (67.5 s [IQR, 49.3-90.0 s] vs 33.0 s [IQR, 23.0-46.0 s], respectively; Mann-Whitney U test, P <.001).

### Treatment efficacy and oncologic outcomes

#### Technical success and tumor volume changes

Immediate post-ablation contrast-enhanced ultrasonography (CEUS) demonstrated complete absence of intratumoral enhancement in all 303 patients, resulting in a technical success rate of 100%.

The median pre-ablation tumor volume was 87.9 mm³ (IQR, 38.-180.4 mm³). The median ablation zone volume at 1 month after RFA was 473.6 mm³ (IQR, 283.0-791.9 mm³). Tumor volume increased significantly at 1 month after ablation compared with baseline volume (P <.001).

**Figure 2:**
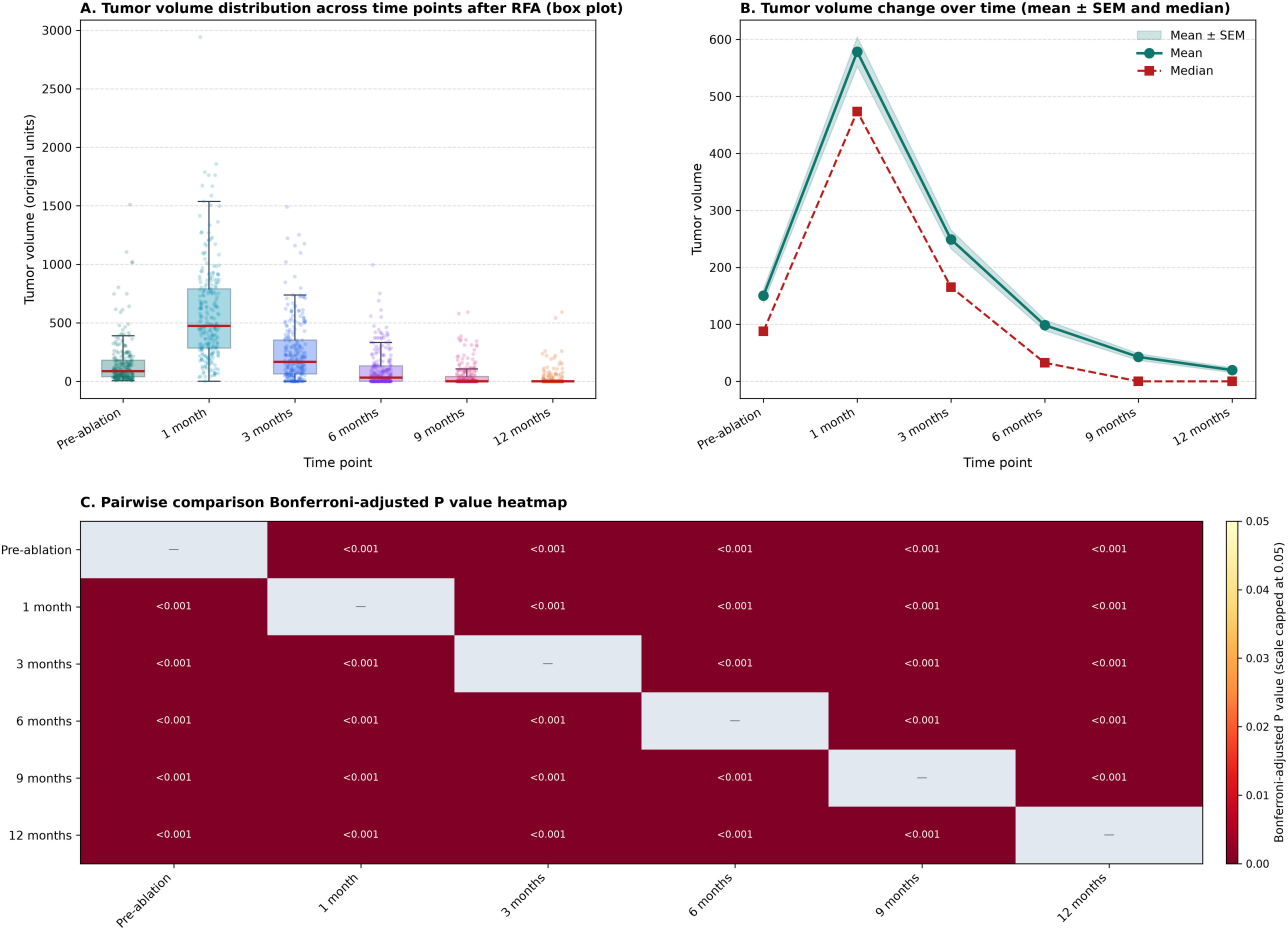
Comparative analysis of tumor volume at each follow-up time point. Asterisks indicate statistically significant differences compared with the 1-month post-ablation volume.

Subsequently, the ablation zone volume decreased progressively over time. Significant reductions were observed at 3, 6, 9, and 12 months compared with the 1-month post-ablation volume (P <.001 for all comparisons) (**Figure 2**).

### Complete tumor disappearance

**Figure 3:**
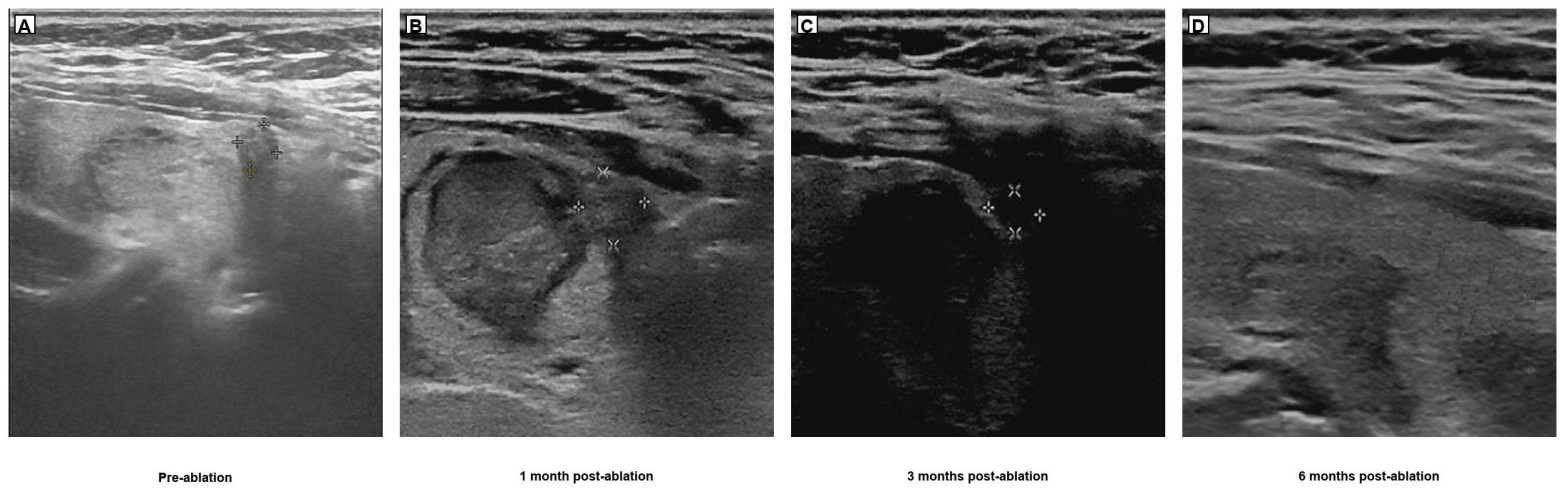
Representative ultrasound follow-up of a capsule-adjacent papillary thyroid carcinoma treated with radiofrequency ablation. **(A)** Pre-ablation image showing the index tumor. **(B)** One month after ablation. **(C)** Three months after ablation, with progressive reduction of the ablation zone. **(D)** Six months after ablation, with complete tumor disappearance of the treated tumor.

During a median follow-up period of 36 months, complete tumor disappearance of the treated tumor was observed in 294 patients (97.0%). A representative case is shown in **Figure 3**.

**Figure 4:**
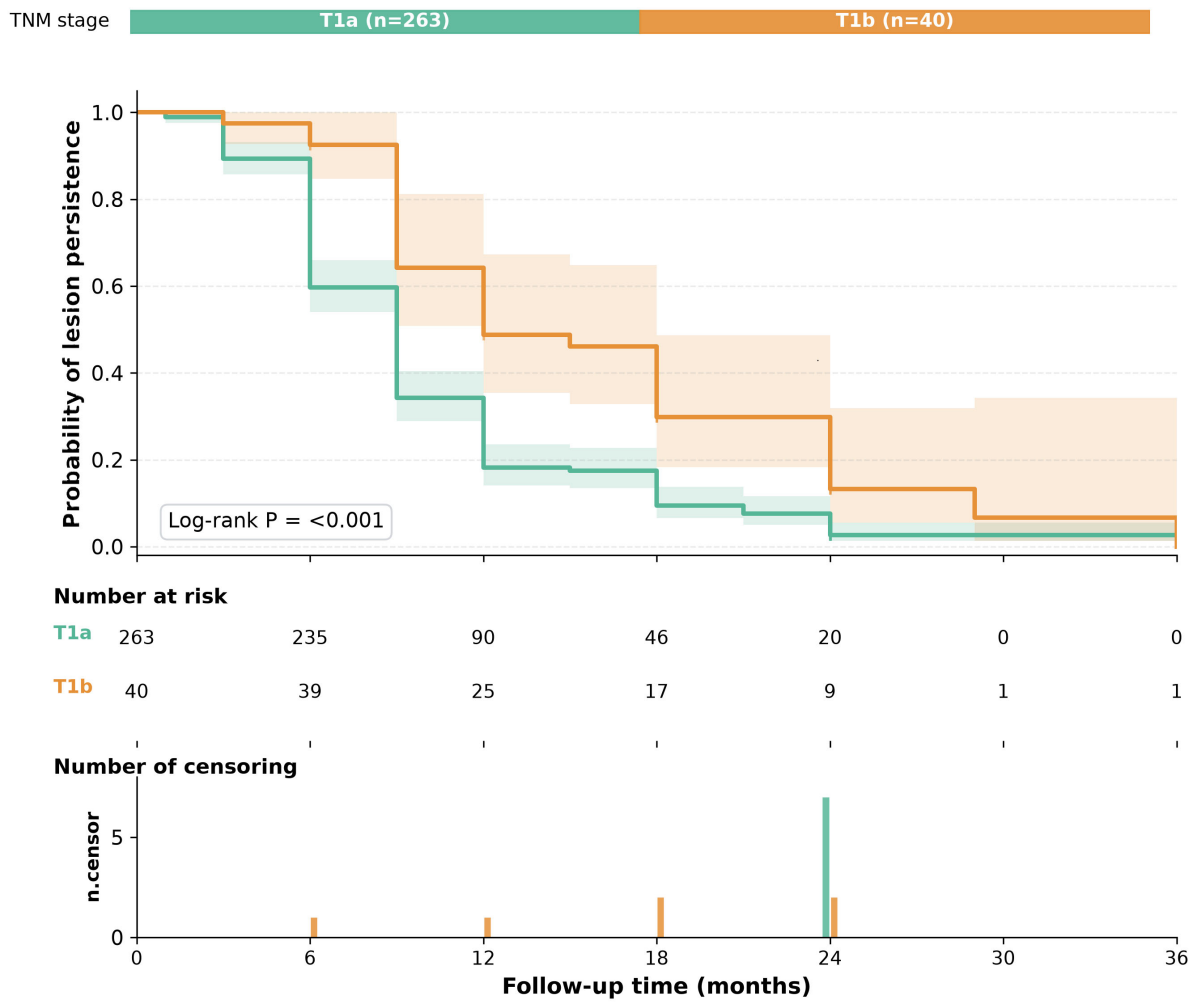
Kaplan–Meier curves of time to complete tumor disappearance after radiofrequency ablation (RFA) in the T1a and T1b groups. The y-axis represents the probability of persistence of the treated lesion. Complete tumor disappearance was defined as the event, whereas patients without complete disappearance at the last follow-up were censored. The median time to complete tumor disappearance was 9 months in the T1a group (green, n = 263) and 12 months in the T1b group (orange, n = 40). The log-rank test showed a significant difference between the two groups (P < 0.001). The numbers at risk and censored observations at each follow-up time point are shown below the plot.

The complete disappearance rate was higher in the T1a group than in the T1b group (98.1% [258/263] vs 90.0% [36/40]), Kaplan-Meier analysis demonstrated a significantly shorter time to complete disappearance in the T1a group than in the T1b group (median, 9 vs 12 months; log-rank P < 0.001) (**Figure 4**).

### Disease progression

During follow-up, disease progression occurred in five patients (1.65%, 5/303). All progression events occurred in patients with T1a tumors.

Among these five patients, four developed newly diagnosed primary thyroid carcinomas, including two ipsilateral and two contralateral tumors. One patient developed ipsilateral cervical lymph node metastasis.

Two patients with disease progression harbored BRAF^V600E^ mutations, whereas three patients were BRAF^V600E^-negative. BRAF^V600E^ mutation status was not significantly associated with disease progression in the overall cohort (P = .99).

### Treatment safety

Procedure-related complications occurred in 10 patients (3.3%, 10/303). All complications presented as transient hoarseness, and all patients recovered spontaneously within 6 months without permanent sequelae.

The incidence of transient hoarseness was significantly higher in the T1b group than in the T1a group (10.0% [4/40] vs 2.3% [6/263], respectively; P = .034).

No other major complications, including permanent vocal cord paralysis, symptomatic hematoma, esophageal injury, or skin burn, were observed (**Table 2**).

**Table 2:** Disease progression and complications in T1a and T1b groups after ablation.

Variable	T1a	T1b
Disease progression	5(1.9%)	0(0)
New tumors		
Location		
ITL	2(0.8%)	0(0)
CTL	2(0.8%)	0(0)
LNMs	1(0.4%)	0(0)
Complications		
Voice hoarseness	6(2.3%)	4(10%)
Hematoma	0(0)	0(0)
Cough	0(0)	0(0)
High fever	0(0)	0(0)

Data are expressed as number of findings, with percentages in parentheses. CTL, contralateral thyroid lobe; ITL, ipsilateral thyroid lobe; LNM, lymph node metastasis.

## Discussion

### Clinical significance of capsule-adjacent PTC

RFA has emerged as an effective minimally invasive treatment for carefully selected patients with low-risk PTC, offering excellent oncologic control while preserving thyroid function and reducing treatment-related morbidity compared with surgery (12, 13). However, evidence regarding tumors immediately adjacent to the thyroid capsule remains limited because these lesions pose unique technical challenges and carry a higher theoretical risk of thermal injury to adjacent critical structures. In this study, we specifically evaluated solitary T1N0M0 PTCs located within 2 mm of the thyroid capsule and demonstrated that ultrasound-guided RFA achieved excellent mid-term outcomes, including a 100% technical success rate, a 97.0% complete tumor disappearance rate, a disease progression rate of only 1.65%, and a low overall complication rate of 3.3%.

A major strength of the present study is its focus on a well-defined high-risk anatomical subgroup. Most previous studies have evaluated capsule-adjacent tumors together with heterogeneous T1 cohorts, making it difficult to determine whether proximity to the thyroid capsule independently influences treatment outcomes. By restricting enrollment to tumors located within 2 mm of the thyroid capsule, our study provides clinically relevant evidence for a population frequently encountered in routine practice but insufficiently investigated.

### Oncologic efficacy and tumor disappearance

The excellent local tumor control observed in our cohort is consistent with previous reports demonstrating durable oncologic outcomes after RFA for low-risk PTC (14–16). Immediate CEUS confirmed complete ablation in all treated lesions, and nearly all tumors disappeared completely during follow-up. These favorable outcomes likely reflect the inclusion of solitary intrathyroidal tumors without imaging evidence of extrathyroidal extension or metastatic disease, together with a standardized treatment protocol that incorporated immediate CEUS to identify residual viable tissue requiring supplementary ablation.

Tumor size appeared to influence treatment response. Complete tumor disappearance occurred significantly later in patients with T1b tumors, and larger tumors generally required longer ablation times and greater cumulative energy delivery. The significantly longer ablation times observed in the T1b group support the interpretation that larger lesions are associated with increased procedural complexity and slower resorption of the ablation zone.

Disease progression was uncommon and occurred in only five patients during follow-up. Four patients developed new primary carcinomas, whereas one developed cervical lymph node metastasis. Because most progression events represented newly developed tumors rather than recurrence within the original ablation zone, these findings likely reflect the multicentric nature of PTC rather than incomplete ablation of the treated lesion, consistent with previous long-term observations after thermal ablation (5, 16).

### Procedural safety and determinants of complications

Procedural safety represents the principal concern when RFA is performed for capsule-adjacent PTC because of the close proximity of the recurrent laryngeal nerve (RLN), trachea, esophagus, and parathyroid glands. Nevertheless, the overall complication rate in our cohort was only 3.3%, with all events consisting of transient hoarseness that resolved spontaneously within six months. No permanent RLN injury, esophageal injury, symptomatic hematoma, skin burns, or other major complications were observed, findings that are consistent with previous large cohort studies and recent meta-analyses demonstrating a favorable safety profile for thyroid RFA when performed by experienced operators using standardized techniques (14, 15).

Although treatment remained safe overall, transient hoarseness occurred significantly more frequently in patients with T1b tumors than in those with T1a tumors. Compared with smaller tumors, T1b lesions required substantially longer ablation times, thereby increasing cumulative thermal exposure to adjacent structures. In addition, nearly half of the tumors were located along the posterior capsule or within the paratracheal region, corresponding to the so-called danger triangle, where the RLN courses immediately adjacent to the thyroid capsule with minimal intervening tissue. All cases of transient hoarseness occurred in these high-risk regions, emphasizing the importance of both tumor size and anatomical location in preprocedural risk assessment.

### Technical considerations for anatomically challenging lesions

Despite these anatomical challenges, permanent nerve injury was not observed in our study, suggesting that meticulous technique can substantially improve procedural safety. Several aspects of our standardized protocol may have contributed to these favorable outcomes. Hydrodissection was routinely performed to establish a stable fluid barrier between the thyroid capsule and adjacent critical structures, thereby reducing thermal transmission during ablation (9, 10). For tumors located within the danger triangle, a lever maneuver was applied when necessary to mechanically displace the lesion away from vulnerable structures. In addition, conservative power settings together with short active-tip electrodes facilitated gradual and predictable thermal expansion.

An additional feature of our protocol was the use of a location-specific ablation strategy. Ablation was intentionally terminated once the transient hyperechoic zone reached the thyroid capsule, whereas tumor margins surrounded by normal thyroid parenchyma were treated with an expanded ablation margin when anatomically feasible. This approach differs from a uniform circumferential expansion strategy and reflects the need to balance oncologic completeness against procedural safety in anatomically constrained lesions.

### Implications, molecular findings, and limitations

These findings suggest that thyroid capsule adjacency alone should not preclude consideration of RFA for carefully selected patients with low-risk PTC. However, tumor size and precise anatomical location should be incorporated into treatment decision-making. In particular, patients with larger tumors located along the posterior capsule or within the paratracheal region should undergo individualized risk assessment and receive appropriate preprocedural counseling regarding the increased likelihood of temporary voice changes.

Another noteworthy finding is the absence of a significant association between BRAF^V600E^ mutation status and disease progression following RFA. Because progression events were infrequent and predominantly manifested as new primary carcinomas rather than local recurrence, the completeness of local tumor ablation may have a greater influence on short- and mid-term outcomes than molecular alterations alone. Nevertheless, these findings should be interpreted cautiously and require validation in larger prospective studies.

Several limitations should be acknowledged. This was a retrospective single-center study, relatively few patients experienced disease progression, and the median follow-up of 36 months provides only mid-term outcome data. In addition, pathological diagnosis was based on FNA rather than surgical specimens, precluding assessment of certain histopathological characteristics such as microscopic extrathyroidal extension, vascular invasion, and occult multifocal disease.

Several limitations should be acknowledged. First, this was a retrospective single-center study, which inevitably introduces the possibility of selection bias despite consecutive patient enrollment. Second, although this represents one of the largest dedicated cohorts of capsule-adjacent PTC treated with RFA, relatively few patients had paratracheal tumors or experienced disease progression, limiting the statistical power of subgroup analyses. Third, the median follow-up of 36 months provides only mid-term outcome data and is insufficient to evaluate very late recurrence or disease-specific survival. Finally, pathological diagnosis was based on fine-needle aspiration rather than surgical specimens, precluding assessment of certain histopathological characteristics such as microscopic extrathyroidal extension, vascular invasion, and occult multifocal disease.

In conclusion, our findings support ultrasound-guided RFA as an effective and generally safe treatment option for carefully selected solitary T1N0M0 PTC located within 2 mm of the thyroid capsule. Rather than thyroid capsule adjacency itself, tumor size, precise anatomical location, and procedural expertise appear to be the principal determinants of treatment safety. Future prospective multicenter studies with longer follow-up and standardized technical protocols are warranted to validate these findings, optimize patient selection, and further define the role of RFA relative to surgery and active surveillance in the management of anatomically challenging low-risk PTC.

## Data Availability

The raw data supporting the conclusions of this article will be made available by the authors, without undue reservation.
